# Spastin Couples Microtubule Severing to Membrane Traffic in Completion of Cytokinesis and Secretion

**DOI:** 10.1111/j.1600-0854.2008.00847.x

**Published:** 2008-10-29

**Authors:** James W Connell, Catherine Lindon, J Paul Luzio, Evan Reid

**Affiliations:** 1 Department of Medical Genetics and Cambridge Institute for Medical Research, University of CambridgeCambridge, UK; 2 Gurdon Institute, University of CambridgeCambridge, UK; 3 Current address: Department of Genetics, University of CambridgeCambridge, UK; 4 Department of Clinical Biochemistry and Cambridge Institute for Medical Research, University of CambridgeCambridge, UK

**Keywords:** abscission, ER-to-Golgi traffic, ESCRT complex, hereditary spastic paraplegia, spastin

## Abstract

Mutations in the gene encoding the microtubule (MT)-severing protein spastin are the most common cause of hereditary spastic paraplegia, a genetic condition in which axons of the corticospinal tracts degenerate. We show that not only does endogenous spastin colocalize with MTs, but that it is also located on the early secretory pathway, can be recruited to endosomes and is present in the cytokinetic midbody. Spastin has two main isoforms, a 68 kD full-length isoform and a 60 kD short form. These two isoforms preferentially localize to different membrane traffic pathways with 68 kD spastin being principally located at the early secretory pathway, where it regulates endoplasmic reticulum-to-Golgi traffic. Sixty kiloDalton spastin is the major form recruited to endosomes and is also present in the midbody, where its localization requires the endosomal sorting complex required for transport-III-interacting MIT domain. Loss of midbody MTs accompanies the abscission stage of cytokinesis. In cells lacking spastin, a MT disruption event that normally accompanies abscission does not occur and abscission fails. We suggest that this event represents spastin-mediated MT severing. Our results support a model in which membrane traffic and MT regulation are coupled through spastin. This model is relevant in the axon, where there also is co-ordinated MT regulation and membrane traffic.

The hereditary spastic paraplegias (HSPs) are a group of genetic neurodegenenerative disorders affecting the neurons of the corticospinal tract. Neuropathologically, HSPs show a length-dependent distal degeneration of the axons of these neurons, causing progressive spastic paralysis affecting the legs (1,2). The HSPs are therefore important models for understanding molecular mechanisms involved in axonal maintenance and degeneration.

Mutations in the *spastin*gene are the most frequent cause of HSP, occurring in approximately 40% of autosomal dominant HSP families (3,4). The mutational spectrum is broad and includes large deletions (sometimes encompassing the entire coding region), suggesting that in many cases the pathological mechanism is haploinsufficiency (5). Missense mutations in the adenosine triphosphatase (ATPase) domain are also common, and these may act through haploinsufficiency or, because spastin forms hexamers, by a dominant-negative effect (6,7).

There are two main spastin isoforms, coded from differing translational initiation sites (Figure 1). Translation beginning at the first ATG results in a 616 amino acid, full-length protein (68 kD), while translation from a second ATG results in a short form (60 kD) that lacks the N-terminal 86 residues of full-length spastin (8). The short form is the most abundant type in brain and spinal cord and in a variety of cell lines (8,9). In most tissues and cell lines studied, full-length spastin is expressed at a low level, although it is enriched in the adult spinal cord (8,10). All spastin mutations identified in families with autosomal dominant inheritance could potentially affect both isoforms, so it is not clear whether one particular form is important for HSP pathogenesis.

**Figure 1:**
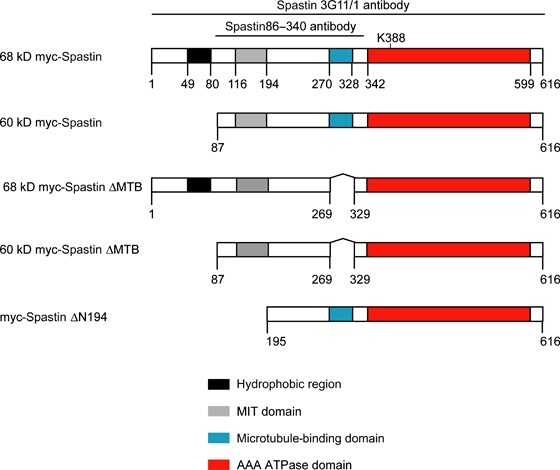
Schematic diagram of spastin's domain structure and constructs used Numbering refers to amino acid position. The regions of the protein against which the antibodies used in the study (3G11/1 and spastin86–340) were raised are shown, as is the position of lysine 388, mutated to arginine in the K388R constructs.

Spastin is an *A*TPases *a*ssociated with diverse cellular *a*ctivities (AAA) protein (3). Extensive evidence indicates that 68 and 60 kD spastin interact with and sever microtubules (MTs) (6,^9^,^11^–16). The mechanism of severing has been clarified recently with the solution of the structure of the *Drosophila*spastin AAA ATPase domain. Hexameric spastin forms a ring with a central pore, into which it is proposed that the C-terminal tail of tubulin is pulled, generating a mechanical force that breaks the MT (7). Consistent with this, endogenous spastin has been reported at regions of active MT regulation, including axonal branches, the distal axon and the midbody during cell division (17,18).

Our previous localization studies with overexpressed 68 kD spastin suggested that spastin's MT-severing activity may be targeted to organelles on the secretory or endocytic pathways (19,20). In addition, it is striking that many of the proteins that interact with spastin have been implicated in membrane traffic, especially at the early secretory pathway or at endosomes (Table 1). One of these membrane-associated binding partners, atlastin, is also an HSP protein, suggesting that spastin's role in relation to membrane traffic may be fundamental to the pathogenesis of the disease. Atlastin is located on the secretory pathway and interacts with a domain within the first 80 amino acids of spastin, so is only able to bind to the 68 kD form (20).

**Table 1:** Verified spastin interactors

Verified spastin interactors	Subcellular location
Atlastin	ER, ERGIC, Golgi (20,21)
CHMP1B	Endosomes (19)
NA14	Centrosome (17)
Reticulon1	ER (22)
ZFYVE27	Endosomes (23)

Spastin's membrane-associated interactors also include the endosomal protein CHMP1B, a protein associated with the endosomal sorting complex required for transport (ESCRT)-III complex (19). Spastin's CHMP1B-binding domain has been narrowed to a region incorporating its MIT (MT-interacting and -trafficking) domain (Figure 1), consistent with the observation that MIT domains of several proteins bind to ESCRT-III members (24). In some cases, the endosomal location of the MIT domain protein requires this interaction (24). ESCRT complexes are required for the formation of, and sorting into, the multivesicular body. In addition, they have an important role in membrane modelling events during cell division, where they are required for the late stage of cytokinesis known as ‘abscission’ or ‘completion’, where the midbody connecting newly divided cells is sealed by addition of new membrane to generate separated daughter cells (25–27). During this process, the ESCRT proteins are located on either side of the densest part of the midbody termed the Fleming body or stembody, sometimes in a double-ring structure. The separation of the daughter cells occurs in association with loss of an abundant parallel array of MTs within the midbody, although the cellular machinery responsible for this MT restructuring has not yet been identified (28,29).

In this paper, we demonstrate that membrane traffic and MT regulation are coupled through spastin. We show that endogenous spastin is present on MTs, the early secretory pathway, endosomes and at the cytokinetic midbody and that its localization to some of these sites is isoform specific. ATPase-defective 68 kD spastin delays traffic of cargo from the endoplasmic reticulum (ER) to the Golgi, while endogenous spastin is required for completion of cytokinesis, where it is necessary for a midbody MT restructuring event that we propose represents spastin-mediated MT severing.

## Results

### Endogenous spastin is located on the early secretory pathway, endosomes and MTs

Previous work has shown that four spastin isoforms [full length (68 kD), exon 4-deleted full length (64 kD), short form (60 kD) and exon 4-deleted short form (55 kD)] are expressed in mammalian cells (8). Using a sensitive novel spastin antibody (spastin86–340), we saw bands corresponding to the size of these isoforms in HeLa, MRC5 human lung fibroblast and NSC34 mouse lower motor neuron neuroblastoma fusion cell lines (Figure S1). In each cell type, 60 kD spastin was strongly expressed, but 68 kD spastin expression was much weaker, as previously reported (8,9).

**Figure 2:**
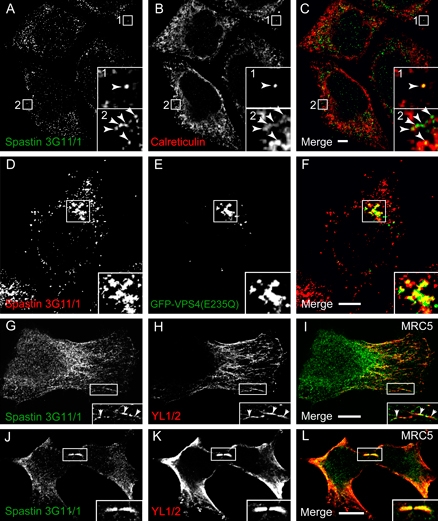
Endogenous spastin's subcellular location A–C) Hela cells labeled with spastin (A) and calreticulin (B) show infrequent colocalized puncta (box 1) and tubules (box 2). D–F) Overexpression of VPS4-E235Q (E) results in redistribution of endogenous spastin (D) to VPS4-E235Q-positive endosomes. G–I) Spastin (G) tubulin (H) colocalization was detected in the cytoplasm of MRC5 cells. The zoomed box shows colocalization on a filament. J–L) Spastin (J) and tubulin (K) showed strong colocalization in the midbody of cells undergoing cytokinesis. In (A–C) and (G–I), arrowheads indicate structures showing co-localisation. In these and subsequent micrographs, right hand panels show the merged images; the colour of each marker in the merged image is shown by the colour of its lettering in the non-merged panels. Scale bars in these and subsequent micrographs = 10 μm. Formaldehyde fixation

Using overexpression systems, we have shown that spastin is present, at least in part, at the ER and endosomes (19,20). To test these findings with the endogenous protein, we examined spastin's subcellular location by immunofluorescence using a commercial antibody that worked well for this application (3G11/1; Figure S1). In general, spastin showed a strong punctate or occasionally filamentous cytoplasmic staining pattern, with significant nuclear staining in some cells (Figure 2A and Figure S1). A small proportion of endogenous cytosolic spastin colocalized with the ER in puncta and tubules (Figure 2A–C). We saw minimal colocalization of spastin and endogenous endosomal markers (Figure S2 and data not shown). However, to reveal any dynamic association between spastin and endosomes, we used dominant-negative VPS4. VPS4 normally removes ESCRT complexes and associated proteins from late endosomes, and the VPS4-E235Q mutant traps on endosomes proteins, including ESCRT complex members, that have a transient localization there (30). We found strong recruitment of endogenous spastin to endosomes on expression of VPS4-E235Q in HeLa and NSC34 cells (Figure 2D–F and Figure S2).

As well as these localizations to membrane compartments, we found steady-state colocalization between spastin and MT markers. This was seen in all cell types examined but was most clearly observed in MRC5 cells, which have a sparse MT network that facilitates morphological analysis (Figure 2G–I) (31). In dividing cells, there was recruitment of spastin to the midbody where it colocalized strongly with MTs in formaldehyde-fixed cells (Figure 2J–L).

In summary, at steady state, spastin colocalized with MTs in the cell body and midbody and, to a much lesser extent, with the ER. In addition, a dynamic pool of cytoplasmic spastin could be strongly recruited to endosomes, from where its removal requires VPS4.

### Spastin's location at the early secretory pathway or endosomes is isoform specific

**Figure 3:**
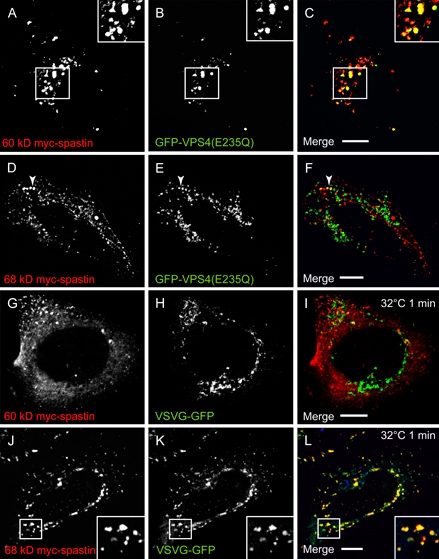
Spastin's recruitment to membrane compartments is isoform specific A–C) Coexpression of 60 kD myc-spastin (A) and VPS4(E235Q) (B) in Hela cells shows strong recruitment of the 60 kD myc-spastin isoform to VPS4(E235Q) endosomes. D–F) Sixty-eight kiloDalton myc-spastin (D) and VPS4(E235Q) (E) coexpressed in Hela cells show minimal colocalization (arrowhead). G–I) Hela cells were transfected with 60 KD myc-spastin (G) and VSVG-GFP (H). VSVG-GFP has just been released from the ER in a synchronized pulse and is concentrated in puncta. There is no colocalization between 60 kD myc-spastin and VSVG-GFP. J–L) In contrast, there is strong colocalization between 68 kD myc-spastin (J) and VSVG-GFP (K), just after VSVG-GFP release in a synchronized pulse from the ER of HeLa cells. Formaldehyde fixation.

We examined whether recruitment of spastin to the midbody, ER or endosomes was isoform specific using epitope-tagged versions of 60 and 68 kD spastin. Both spastin isoforms could be recruited to the midbody, although this recruitment appeared stronger with the 60 kD isoform (Figure S3). At endosomes, there was strong recruitment of transiently expressed 60 kD myc-spastin to VPS4-E235Q puncta (Figure 3A–C). This recruitment did not require spastin's ATPase activity because a disease-associated mutant version of 60 kD spastin that is unable to hydrolyze ATP (spastinK388R) was also recruited to VPS4-E235Q endosomes (data not shown). However, only a small proportion of the puncta seen on transient expression of 68 kD myc-spastin colocalized with VPS4-E235Q (Figure 3D–F). These data indicate that the 60 kD form of spastin is the main endosomal form.

To study the relationship between 60 and 68 kD myc-spastin and the early secretory pathway, we used vesicular stomatitis virus G (VSVG)-green fluorescent protein (GFP) (GFP-tagged temperature-sensitive mutant of the vesicular stomatitis viral glycoprotein) assays. At a temperature of 40°C, this protein is retained in the ER, while on shifting to a permissive temperature of 32°C, it exits the ER in a synchronized pulse and is transported by the ER-to-Golgi intermediate compartment (ERGIC) to the Golgi (32). We found minimal colocalization between 60 kD myc-spastin and ER markers or VSVG-GFP at early or later time-points (Figure 3G–I, Figure S4). In contrast, at early time-points (up to 5 min) after release of VSVG-GFP from the ER, we found almost complete colocalization between 68 kD myc-spastin and VSVG-GFP puncta (Figure 3J–L). At later time-points, this colocalization was lost as the VSVG-GFP transited to the Golgi. These results indicate that 68 kD spastin is principally located on the early secretory pathway (ER and ERGIC), whereas 60 kD spastin is not. The small amount of endogenous spastin that we saw on the ER (Figure 2A–C) likely represents 68 kD spastin there.

We next sought to identify functions of spastin at the membrane sites where we had identified it.

### ATPase-defective 68 kD spastin delays ER-to-Golgi traffic

We first examined the functional role of 68 kD spastin at the early secretory pathway. Because spastin regulates MTs, we examined rates of transport of VSVG-GFP between the ER and the Golgi apparatus, as this depends on MT-based vesicular transport (33). We used HeLa cells, as they express 68 kD spastin (8) and as this assay has been well characterized in them. To model the effects of a disease-associated missense mutant, we began by comparing the effects of wild-type 68 kD myc-spastin and ATPase-defective 68 kD myc-spastinK388R on the trafficking of VSVG-GFP after its release in a pulse from the ER.

**Figure 4:**
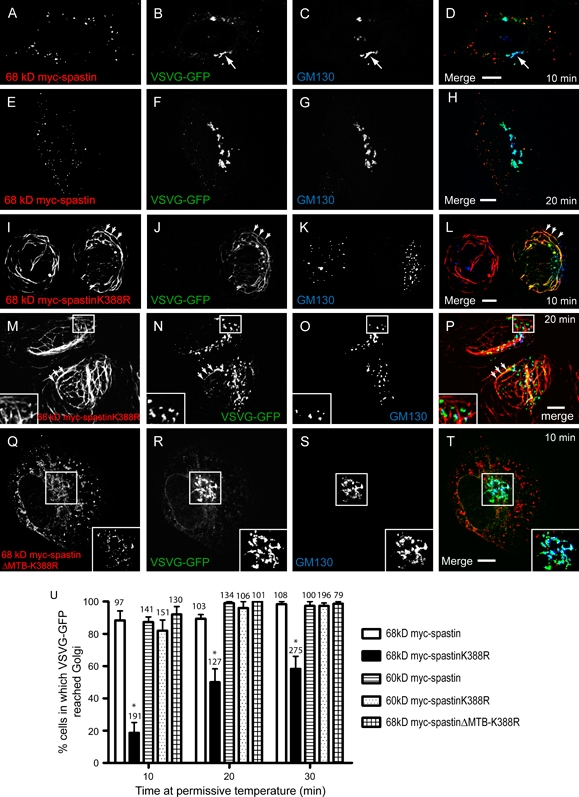
Sixty-eight kiloDalton spastinK388R delays ER–Golgi traffic of VSVG-GFP Hela cells were cotransfected with VSVG-GFP and 68 kD myc-spastin (A–H), 68 kD myc-spastin K388R (I–P) or 68 kD myc-spastinΔMTB-K388R (Q–T). VSVG-GFP was then released in a pulse from the ER. A–H) In cells expressing 68 kD myc-spastin (A and E), VSVG-GFP fluorescence (B and F) had left the ER and was strongly associated with the Golgi marker GM130 (C and G) at 10 min (A–D, arrow) and 20 min (E–H) post release. I–L) In contrast, at 10 min post release in cells expressing 68 KD myc-spastin K388R (I), VSVG-GFP (J) remained predominantly in the ER on myc-spastinK388R-positive MT bundles (arrowheads) and only a few VSVG-GFP-positive vesicles had left the ER. In most cells, VSVG-GFP showed minimal or no colocalization with the Golgi (K). M–P) At 20 min, although some VSVG-GFP (N) was retained on the myc-spastinK388R-positive MT bundles (arrowheads), most had emerged. However, much of the VSVG had not reached the Golgi (O), as demonstrated by the presence of green vesicles in the merged image (P). The Golgi often appeared fragmented in cells expressing 68 kD myc-spastinK388R (K and O). Q–T) Ten minutes after VSVG-GFP release in cells expressing 68 kD myc-spastinΔMTB-K388R (Q), VSVG-GFP (R) showed strong colocalization with the Golgi (S). U) Quantification of these results showed that, at each time, VSVG-GFP had reached the Golgi in a smaller percentage of cells transfected with 68 kD myc-spastinK388R (*n*= 3 experiments), compared with other spastin constructs tested (p < 0.002, two-way anova; *n*= 3 for each spastin construct). The total number of cells counted at each time is indicated above the relevant bar. Error bars = SEM. Formaldehyde fixation.

In cells transfected with wild-type 68 kD myc-spastin, just after release from the restrictive temperature, VSVG-GFP colocalized strongly with the ER marker calreticulin, as expected (Figure S5). At 10 min (Figure 4A–D) and 20 min (Figure 4E–H) after release, VSVG-GFP showed strong and increasing colocalization with the Golgi marker GM130. At 60 and 120 min, most of the VSVG-GFP had left the Golgi and was associated with the plasma membrane (data not shown and Figure S5). This pattern of VSVG-GFP traffic was identical to that seen in cells transfected with VSVG-GFP alone (data not shown).

Expression of 68 kD spastinK388R results in a cellular phenotype of thickened and bundled MTs that closely associate with and redistribute the ER (Figure S5) (20). In contrast to the results with wild-type 68 kD spastin, in cells expressing this protein, ER-to-Golgi traffic of VSVG-GFP was considerably delayed. Thus, after 10 min, most VSVG-GFP remained in the ER, colocalized with the spastinK388R filaments and minimal colocalization with GM130 was seen (Figure 4I–L). At 20 min, some of the VSVG-GFP remained in the ER and the majority of it had not yet reached the Golgi (Figure 4M–P). After 60 min, we saw strong colocalization between VSVG-GFP and GM130 in most cells (data not shown), and by 120 min, much was still in the Golgi (Figure S5). Quantification of VSVG-GFP traffic to the Golgi revealed that, compared with cells transfected with wild-type 68 kD myc-spastin, VSVG-GFP had reached the Golgi in a significantly smaller percentage of 68 kD spastinK388R-transfected cells at 10, 20 or 30 min post VSVG-GFP release (p < 0.002, two-way anova; Figure 4U).

We examined whether expression of 60 kD myc-spastin or 60 kD myc-spastinK388R affected ER-to-Golgi traffic of VSVG-GFP and found no delay (Figure S5 and Figure 4U). Even in 60 kD myc-spastinK388R-transfected cells with obvious MT bundling, the transport of VSVG-GFP from the ER to the Golgi did not differ from untransfected cells (Figure S5), suggesting that 60 kD spastin targets a differing MT population to 68 kD spastin.

We also examined the effects of depletion of spastin by small interfering RNA (siRNA). However, we found no effects on VSVG-GFP trafficking, perhaps because siRNA-mediated depletion of 68 kD spastin seemed to be relatively inefficient compared with the 60 kD form (Figure S1), so sufficient 68 kD spastin may have remained.

### The ER-to-Golgi traffic defect seen with 68 kD spastinK388R requires the MTB domain

Because transport of VSVG-GFP from the ER to the Golgi uses MTs (34), we examined whether the ER-to-Golgi traffic delay seen with myc-spastinK388R was dependent on its interaction with MTs. We generated a construct (termed 68 kD myc-spastinΔMTB-K388R; Figure 1) encoding 68 kD myc-spastinK388R but deleted for a MT-binding (MTB) domain lying between residues 270 and 328 that has recently been shown in cells and *in vitro*to be sufficient for spastin's MT association and necessary for MT severing (6). Expression of this protein caused no obvious MT phenotype (data not shown), although it did colocalize strongly with the ER marker calreticulin (Figure S6). VSVG-GFP trafficking in cells expressing this construct was not delayed, with VSVG-GFP showing strong colocalization with Golgi markers from 10 min after release from the ER (Figure 4Q–U).

### Spastin localizes to midbody double-ring structures

We next turned our attention to 60 kD spastin. Sixty kiloDalton spastin contains an MIT domain that interacts with the ESCRT-III protein CHMP1B (19). Because spastin localizes to endosomes and to the midbody, we examined whether it had a role in the known functions of the ESCRT machinery at these sites. We first examined whether depletion of spastin affected the endosomal degradation of the epidermal growth factor receptor (EGFR) but found no significant effect of spastin depletion on EGFR degradation (Figure S7), suggesting that spastin's function is not necessary for degradation of this cargo in HeLa cells.

**Figure 5:**
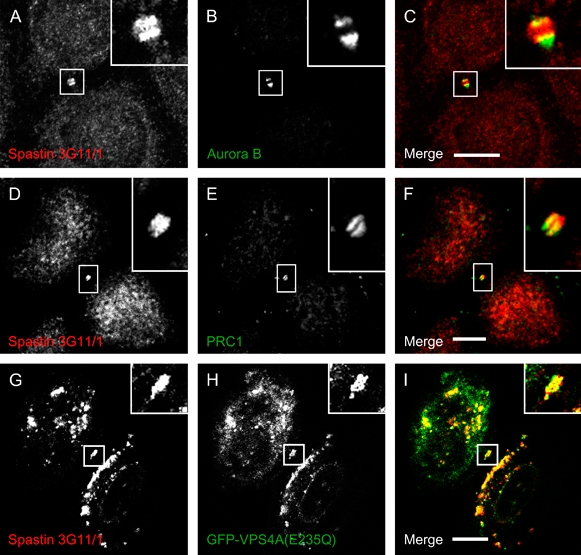
Spastin localizes to double-ring structures in the midbody of methanol-fixed cells A–F) In HeLa cells fixed with methanol, endogenous spastin (A and D) colocalizes with the midbody markers Aurora B (B) and PRC1 (E). Note the double-ring appearance of spastin on either side of the stembody, most obvious in (A). G–I) Spastin (G) also colocalizes with GFP-VPS4A(E235Q) (H) in double-ring structures in the midbody, in methanol-fixed HeLa cells.

We then examined the role of spastin at the midbody during cell division, first examining in more detail the localization of spastin at this site. Following formaldehyde fixation, spastin appears to be present throughout the midbody, where it strongly colocalizes with MTs (Figure 2J–L). However, following methanol fixation, while we still saw some labelling of midbody MTs (data not shown), spastin signal was concentrated in a double-ring structure around the stembody. This structure may have become more prominent after methanol fixation because it was revealed by removal of soluble spastin or because methanol fixation is more favourable to capturing short-lived interactions or revealing epitopes hidden in complex structures. The spastin double-ring structure partially colocalized with the midbody markers Aurora B and PRC1 (Figure 5A–F) (35,36). This double-ring localization was strikingly reminiscent of the appearance described for VPS4 and some ESCRT proteins in the midbody (26,27), and indeed, the spastin double-ring structures showed strong colocalization with VPS4-E235Q (Figure 5G–I), suggesting that they represent spastin in association with ESCRT proteins.

### The MIT domain is required for spastin's recruitment to the midbody

**Figure 6:**
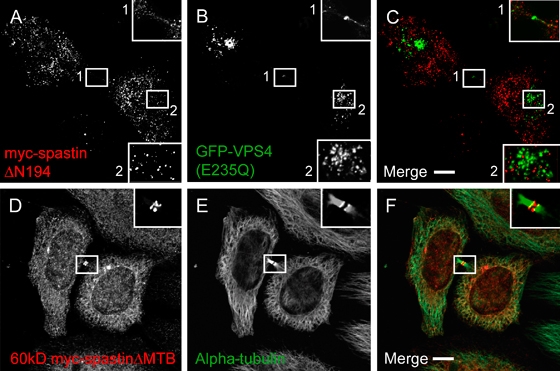
The MIT domain but not the MTB domain is required for recruitment of spastin to the midbody A–C) Myc-spastinΔN194 (A) was not recruited to VPS4(E235Q) structures (B) in the cell body or midbody (box). D–F) 60 kD myc-spastinΔMTB (D) is expressed in a double-ring structure on either side of the stembody, but unlike wild-type 60 kD myc-spastin (Figure S3), does not colocalize with alpha-tubulin (E) throughout the intercellular bridge. Formaldehyde fixation.

The midbody protein CEP55 recruits the ESCRT-1 protein TSG101 and the ESCRT-associated protein Alix to the midbody. Alix in turn recruits ESCRT-III proteins to the midbody, and this recruitment is essential for normal abscission (26,27). Mammalian spastin's MIT domain is not necessary for its interaction with MTs (6) but does interact with the ESCRT-III protein CHMP1B (19). We therefore investigated whether this domain is required for recruitment of spastin to the midbody. We expressed a form of spastin deleted for the N-terminal region and MIT domain (myc-spastinΔN194; Figure 1) and found no recruitment of this construct to the midbody (Figure 6A–C). In addition, we found no recruitment of myc-spastinΔN194 to VPS4-E235Q endosomes in the cell body (Figure 6A–C).

We then examined whether spastin's ability to bind to MTs affected its localization at the midbody. We generated a construct (60 kD myc-spastinΔMTB; Figure 1) encoding residues 87–616 of spastin but deleted for the MTB domain. Under conditions of formaldehyde fixation, deletion of the MTB domain did not prevent recruitment of spastin to the midbody but, compared with wild-type 60 kD myc-spastin (Figure S3), did alter the pattern of its distribution within the midbody. Instead of colocalizing with MTs throughout the intercellular bridge, it was found in a double-ring structure on either side of the stembody that showed only partial colocalization with MTs (Figure 6D–F). These structures were very similar to those found with endogenous spastin after methanol fixation, suggesting that methanol fixation reveals a pool of midbody endogenous spastin that is not associated with MTs.

We conclude from these experiments that spastin's recruitment to the midbody requires the MIT domain but not the MTB domain.

### Spastin is required for abscission

**Figure 7:**
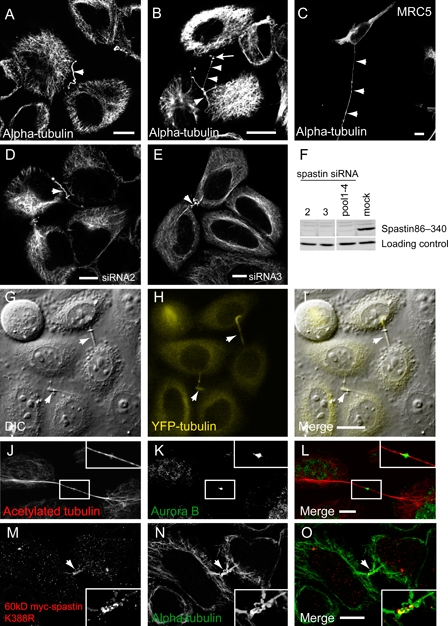
Spastin depletion causes the appearance of MT-filled intercellular bridges A–C) Hela (A and B) and MRC5 (C) cells were labeled with alpha-tubulin following spastin depletion with pooled siRNA oligonucleotides 1–4. Note long intercellular bridges (arrowheads) that were sometimes very convoluted (B). Alpha-tubulin-labeled puncta were often seen in association with these bridges (arrow in B). D and E) The intercellular bridges (arrowheads) were also seen following spastin depletion using two individual spastin siRNA oligonucleotides. Successful spastin depletion in these experiments is verified in (F). G–I) The intercellular bridges (arrowheads) typically joined two cells, as shown in DIC image (G) of YFP–tubulin (H)-expressing HeLa cells depleted of spastin. J–L) Some of the intercellular bridges had the appearance of very elongated midbodies, which labeled with midbody markers [e.g. aurora B; (K)] as well as with MT markers (J). M–O) Hela cells transfected with 60 kD myc-spastinK388R (M) and labeled for alpha-tubulin (N) also displayed similar intercellular bridges (arrowhead). Formaldehyde was used in fixed preparations except (J–L) where methanol was used.

We next investigated whether depletion of spastin caused abnormalities in cytokinesis. In HeLa and MRC5 cells, spastin depletion using an siRNA pool caused a proportion of cells to be connected by extended tubular structures that labeled strongly with antibodies to acetylated, tyrosinated or total tubulin (Figure 7A–C). These structures were long and often convoluted and were sometimes accompanied by puncta that labeled strongly with MT markers (Figure 7B). We also saw them when spastin was depleted using four independent siRNA oligonucleotides, strongly suggesting that they were not the result of an off-target effect (Figure 7D–F and data not shown). Typically, the tubules connected two cells (Figure 7G–I), and sometimes, they resembled elongated midbodies. In these cases, they labeled with midbody markers, indicating that they were elongated versions of the intercellular bridges that form between daughter cells following nuclear division (Figure 7J–L). We saw similar intercellular tubules in a proportion of cells expressing ATPase-defective 60 kD myc-spastinK388R (Figure 7M–O) but not 68 kD myc-spastinK388R.

**Figure 8:**
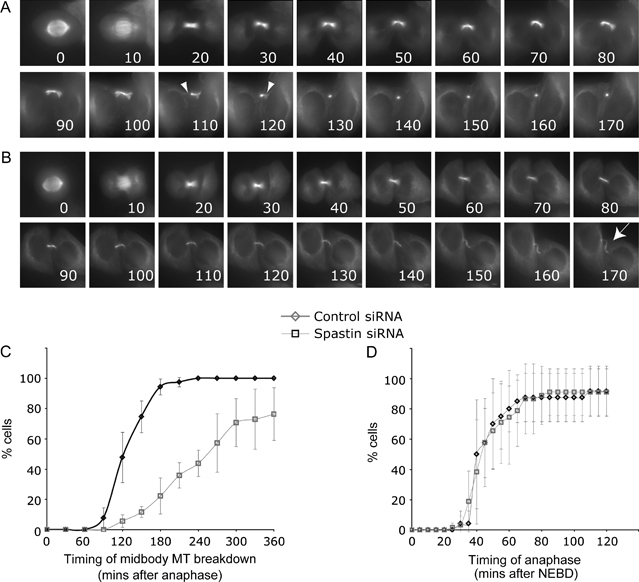
Spastin is required for completion of cytokinesis A) Time -lapse series of control glyceraldehyde-3-phosphate dehydrogenase (GAPDH) siRNA-treated cells, showing the disruption of MTs in the intercellular bridge, on either side of the stembody (arrowheads) at approximately 2 h after anaphase. Times after anaphase shown in min. B) Time-lapse series of YFP–tubulin cells depleted of spastin. The MT disruption event does not occur. Cells remain attached by a microtubule-rich intercellular bridge (arrow). C) Cumulative timing of MT disruption event shown in (A). In control cells, the time from anaphase to MT disruption was measured. In spastin-depleted cells, where this event is delayed or does not occur, it was often not possible to measure (e.g. when it was after the end of filming). We plotted minimum timings for these cells, underestimating the effect of spastin depletion. Control cells, *n*= 57; spastin-depleted cells, *n*= 64, obtained from four independent experiments. p < 0.0001 (Student's *t*-test). D) Cumulative timing of anaphase onset in YFP–tubulin cells. Time from nuclear envelope breakdown (NEBD) to separation of duplicated chromosomes at anaphase was measured. Control cells, *n*= 21 (obtained from four experiments); spastin-depleted cells, *n*= 41 (obtained from three experiments). Error bars = SDs.

These results strongly suggested that spastin is required for completion of a late stage in cytokinesis. We used time-lapse microscopy of HeLa cells stably expressing yellow fluorescent protein (YFP)–tubulin to verify this. In control cells, the midbody MTs that extend from each daughter cell into the intercellular bridge abruptly break down approximately 2 h after anaphase onset (Figure 8A and Video S1) in an event that precedes the final abscission event. In some cells, we saw constriction of the intercellular bridge immediately preceding the loss of midbody MTs (Video S1), consistent with previous reports (28,37). In contrast, in spastin-depleted cells, this MT disruption event was severely delayed or did not occur (Figure 8B,C and Video S1). Instead, the intercellular bridge became progressively thinner and fainter but persisted for many hours, often becoming overextended to resemble the structures that we had seen by immunofluorescence (Figure 7A). We saw these effects both when spastin was depleted using a pool of siRNA oligonucleotides (Figure 8B,C and Video S1) and when each oligonucleotide was used independently (Figure S8). Although abscission clearly failed in these cells, we did not see re-fusion of daughter cells, and we only saw a slight increase in multinucleate cells in fixed preparations [ratio of nuclei/cells: mock = 1.02 (*n*= 224 cells), spastin knock down = 1.05 (*n*= 213 cells)]. Therefore, this failure in abscission is distinct from that occurs when MTs are not properly organized in the midbody, for example following depletion of the MT-bundling protein PRC1 (38), and it seems likely that spastin is not required for midbody organization or function until late in cytokinesis. Spastin plays a role in mitosis in *Drosophila*, where it contributes to the rapid MT dynamics of the mitotic spindle (39). However, we were unable to measure any convincing effect of spastin depletion on the timing of mitosis up to anaphase (Figure 8D and Figure S8), arguing that if spastin plays a similar role in human cells, either this is not essential for mitotic spindle dynamics or the small amounts of spastin left after depletion are sufficient to carry out this function.

We concluded that spastin plays an essential role in causing a MT disruption event that is necessary for completion of the abscission stage of cytokinesis of human cells.

## Discussion

Spastin is a MT-severing enzyme that has a key role in the pathogenesis of HSP. Not only are mutations in the *spastin*gene the most frequent cause of HSP but it also directly interacts with another HSP protein, atlastin (20,21). Elucidating its function is therefore crucial to understand HSP pathogenesis. Its study is also of general cell biological importance because it may reveal mechanistic insights into cellular processes in which MT severing plays a role.

This study provides the first direct evidence that endogenous spastin can be recruited to membrane traffic pathways. Taken together with our previous studies (19,20), our results show that endogenous spastin is located at endosomes and at the early secretory pathway and that recruitment to these sites is isoform specific. In the present study, we found that 60 kD spastin was recruited to endosomes but not the secretory pathway, while 68 kD spastin is mainly located in the ER and very early ER-to-Golgi transport compartments. Our findings therefore suggest that spastin is a MT-severing enzyme that is recruited to different sites on membrane traffic pathways.

What factors might be responsible for recruiting spastin to its different sites of action? While spastin's C-terminal half contains the AAA domain crucial for MT severing, its N-terminal half mediates interactions with at least five other binding partners (Table 1). Its localization to the early secretory pathway clearly depends on the N-terminal 86 residues of 68 kD spastin, which the 60 kD form lacks. This localization may be mediated by spastin's interaction with the ER protein reticulon1 (22). The HSP protein atlastin is present on the early secretory pathway and is able to bind to 68 kD, but not 60 kD, spastin (20,40). In neuronal cells, where it is highly expressed, atlastin binding could be an additional determinant of 68 kD spastin's recruitment to the secretory pathway. Spastin also contains an MIT domain that binds to the ESCRT-III protein CHMP1B (19). Deletion of spastin's MIT domain prevented its recruitment to endosomes, suggesting that its localization to this site is mediated by MIT–ESCRT-III interactions. Thus, spastin's localization may be influenced by interactions with a variety of adaptor proteins.

With the caveat that our results relied on an overexpression system, we found that 68 kD spastin functions in ER-to-Golgi traffic because expression of ATPase-defective 68 kD spastinK388R significantly delayed ER-to-Golgi traffic of VSVG-GFP. This effect depended on its ability to interact with MTs by the MTB domain. Budding from ER exit sites and transport from ER-to-Golgi is dependent on MT dynamics and motility, so we speculate that disruption of ER-associated MTs caused by spastin mutation affects one or both of these processes (34,41). The notion that 68 kD spastin is involved in formation and transport of ER-to-Golgi carriers fits well with reports that expression of atlastin mutants disrupts vesicular budding from the ER and traffic of ER-to-Golgi transport intermediates (42). Although a recent publication has suggested that expression of atlastin mutants does not affect VSVG-GFP traffic (43), this publication did not report on early time-points after VSVG-GFP release from the ER when we found the most significant effects of spastin mutation.

The observation that 60 kD spastin can localize to endosomes adds it to the group of HSP proteins that localize to this site, which includes NIPA1, maspardin and spartin (44–46). Although we did not find effects of spastin depletion on endosomal EGFR degradation, we identified a role for mammalian spastin in another process that requires the ESCRT machinery, cytokinesis. During cytokinesis in animal cells, contraction of an equatorial actomyosin ring drives cleavage of the cell and compaction of bundled, antiparallel MTs into a structure termed the midbody (47). The MTs are embedded in the densest part of the midbody known as the stembody or Fleming body. The daughter cells remain connected by a thin, MT-packed bridge of plasma membrane-bound cytoplasm for some time after cleavage. The midbody is essential for the formation and maintenance of this intercellular bridge as well as for its final resolution (47). Consistent with previous reports, we found that endogenous spastin localizes to the midbody (17).

Early description of the completion of cytokinesis described constriction of the intercellular bridge that occurs before the final separation of daughter cells (37). More recently, imaging of MTs in live cells has shown that this corresponds to loss of MTs in the intercellular bridge, although the machinery required has not been identified (28,29). In this study, we have shown that, in human cells, the disruption of MTs that accompanies abscission requires spastin. Although gradual thinning of the intercellular bridge occurred in cells lacking spastin, the MT disruption event in the half-bridges on either side of the stembody that normally accompanies abscission did not occur. Instead, midbody breakage failed, and daughter cells remained attached by thin, frequently very extended intercellular bridges for many hours. These results strongly suggest that spastin is part of the machinery of abscission, and we speculate that the MT disruption event, which appears to be essential for this process, represents spastin-mediated severing.

The final step in abscission also requires resolution of the plasma membrane between the daughter cells, and membrane traffic machinery is necessary for this (29,^48^,49). Of particular interest in the context of spastin's interaction with the ESCRT-III complex-associated protein CHMP1B is the role of ESCRT-I and ESCRT-III proteins and the ESCRT-associated protein ALIX. Depletion of ALIX, abrogation of its interaction with the ESCRT-III protein CHMP4 or expression of dominant-negative VPS4-K173Q, which traps ESCRT proteins on endosomes and disrupts their function, all result in a failure of abscission accompanied by subsequent re-fusion of daughter cells and a consequent high incidence of multinucleation (26,27). Although final cleavage of the midbody plasma membrane failed in spastin-depleted cells, the effect of spastin depletion was different to that seen with manipulations of the ESCRT machinery because there was no significant increase in multinucleated cells in fixed preparations, and no re-fusion of daughter cells, even after extended periods of time-lapse microscopy. It is possible that the point at which spastin is required in abscission is late and beyond the time at which daughter cells are capable of re-fusing, although we cannot exclude the possibility that spastin may play an additional role in re-fusion of daughter cells after failure to resolve the plasma membrane. Nevertheless, we conclude that spastin is required for resolution of the midbody plasma membrane.

The ESCRT and VPS4 proteins involved in cytokinesis localize to a double-ring structure on either side of the stembody (27). Formaldehyde fixation revealed endogenous spastin throughout both sides of the midbody, where it colocalized with MTs. However, after methanol fixation, double-ring structures surrounding the stembody, and strikingly similar to those seen with some ESCRT proteins, were more prominent. These double-ring structures showed only limited colocalization with MTs, and similar structures were seen on expression of MTB domain-deleted spastin. When considered with the strong colocalization that we saw in the midbody between endogenous methanol-fixed spastin and VPS4-E235Q, this suggests that the double-ring spastin labelling represents a non-MT-associated pool that is interacting with ESCRT complex members. The ESCRT-III-interacting MIT domain was required for spastin to be located in the midbody. We therefore suggest that spastin is positioned in the midbody by the ESCRT-related cytokinetic machinery, where it then interacts with MTs and carries out a MT-severing function crucial for the completion of abscission. The MTB domain is likely to be essential for this function because although it is not required for spastin to localize in the midbody, it is necessary for MT severing (6) and for spastin to colocalize with midbody MTs.

What is the relevance of our findings to spastin's role in the axon? Recent work indicates that spastin is involved in promoting axonal branching (18). Axonal branching requires delivery of new membrane to the branch site, and early studies showed that MT disruption in the area of the new branch point is necessary and sufficient to promote delivery of membrane-bound cargoes that can insert membrane into the growing branch (50). It has been suggested that new membrane required for axonal branching is derived from a specialized endosomal compartment (51). Because expression of spastin in axons results in decreased MT mass and formation of abundant short MTs, it is likely that the increased branching seen on expression of spastin is as a result of its MT-severing activity (18). Thus, axonal branching resembles the other situations in which we have found spastin because it requires closely co-ordinated membrane traffic and MT regulation. Further work will be required to identify the precise relationship between spastin and membrane traffic compartments in axons. However, based on analogy with spastin's recruitment to endosomes and to the midbody, we speculate that spastin may be concentrated at axonal branch points and the growth cone following recruitment by membrane-associated adaptors, perhaps including atlastin and ESCRT-III proteins.

Although a model for spastin's role in developing axons is emerging, it is not yet clear how this might relate to the axonal degeneration seen in HSP, which cannot involve defects of axonal branching or growth. However, the relationship that we have identified between spastin and membrane traffic processes, against the background of numerous other HSP gene products being involved in endocytosis or secretion, points to the likelihood that this will involve failure of membrane traffic-related functions.

## Materials and Methods

### Constructs

A schematic diagram of the constructs used is shown in Figure 1. Synthesis of pcDNA3.1(+) mammalian expression plasmids containing 68 kD wild-type, 68 kD myc-spastinK388R and myc-spastinΔN194 has been described previously (19,20). Sixty kiloDalton (deleted for the N-terminal 86 amino acids) myc-spastin and myc-spastinK388R were made from the full-length constructs using Phusion™ Site-Directed Mutagenesis (Finnzymes), according to the manufacturer's instructions. MT-binding-domain-deleted (ΔMTB, lacking amino acids 270–328) versions of 60 and 68 kD myc-spastin were made in the same way. Constructs were sequence verified before use. The temperature-sensitive ts045 VSVG-GFP construct was a kind gift from Rainer Duden (University of London), and the GFP-VPS4E235Q construct was a kind gift from Paul Whitley (University of Bath).

### Antibodies

Rabbit polyclonal anti-spastin86–340, used for immunoblotting of spastin, was raised (Harlan SeraLabs) against a glutathione S-transferase fusion protein that we synthesized incorporating residues 86–340 of spastin. Mouse anti-spastin monoclonal antibody (3G11/1), used for immunoblotting and immunofluorescence, was obtained from Santa Cruz Biotechnology. Mouse monoclonal anti-myc antibody (clone 4A6) was obtained from Upstate. Rabbit polyclonal anti-GFP (6556), rabbit polyclonal anti-aurora B and rat polyclonal anti-tyrosinated tubulin (YL1/2) were obtained from Abcam. Rabbit polyclonal anti-myc antibody (A14) and rabbit anti-EGFR (1005) antibody were obtained from Santa Cruz Biotechnology. Rabbit polyclonal anti-calreticulin antibody was obtained from Calbiochem. Mouse monoclonal anti-GM130 antibody was obtained from BD Transduction laboratories. Mouse monoclonal alpha-tubulin (clone DM1A), acetylated tubulin (clone 6-11B-1) and rabbit polyclonal anti-actin antibodies were obtained from Sigma. Rabbit polyclonal anti-PRC1 was a kind gift of Tony Hunter (San Diego). Rabbit polyclonal mannose 6 phosphate antibody was produced as previously described (52). Peroxidase-conjugated secondary antibodies for western blotting were obtained from Sigma. Alexafluor-488-, Alexafluor-568- and Alexafluor-647-labeled secondary antibodies for immunofluorescence were obtained from Molecular Probes.

### Cell culture

HelaM cells were maintained in DMEM containing 10% (v/v) FBS, 100 U/mL penicillin, 100 μg/mL streptomycin and 2 mml-Glutamine (Sigma). HeLa cells stably expressing YFP-tagged tubulin for time-lapse analysis were additionally cultured in the presence of 500 μg/mL Geneticin (Invitrogen). For time-lapse microscopy, cells were seeded onto glass bottom dishes (WillCo Wells BV). DMEM was replaced with Leibovitz's L-15 medium (Invitrogen) before filming.

### Transfection and immunofluorescence

For siRNA transfections, a double-hit knock down protocol was used, as previously described (53). Briefly, 1 × 10^5^ HeLaM cells (54) were plated in a well of a six-well plates and subsequently transfected on two occasions, 48 h apart, using Oligofectamine transfection reagent (Invitrogen), according to the manufacturer's instructions. For DNA transfections, HeLaM cells were plated onto poly-l-lysine (Sigma)-coated coverslips and transfected 24 h later using Effectine® transfection reagent (Qiagen). Twenty-four hours post transfection coverslips were directly processed for immunofluorescence microscopy or first used for ts045 VSVG-GFP transport assays. For immunofluorescence, typically, cells were fixed at room temperature in 4% (v/v) formaldehyde in PBS and permeabilized in PBS containing 0.1% (v/v) Triton-X-100 (Sigma). For certain markers (Aurora B and PRC1), cells were fixed and permeabilized in methanol. Coverslips were incubated in blocking buffer containing 10% (v/v) FBS in PBS for 30 min before incubation with specific monoclonal or polyclonal antibodies for 1 h. Coverslips were washed several times in blocking buffer before incubation with secondary antibody for 1 h. Following this final incubation, coverslips were washed several times in blocking buffer, followed by subsequent washes with PBS and distilled water before being mounted on glass slides with anti-fade Gold mounting medium (Invitrogen). Slides were analysed at room temperature with a Zeiss 510 Meta confocal microscope (×63 oil immersion objective) with LSMImage analysis software. Images were subsequently processed using Adobe Photoshopand Illustrator.

### Spastin siRNA oligonucleotides

siRNA oligonucleotides to spastin were obtained from Dharmacon Inc. (Perbio Science UK). Human spastin individual siGENOME duplexes (SPG4) (D-014070-01 – 04; siRNA1 5′- UUAUAGAAGGUUGAAGUUCUU; siRNA2 5′- UCAUUAUAGACGUCCGUUUUU; siRNA3; 5′- UAAACUUGCAGCACUUAUAUU; siRNA4 5′- UUAGCCAGCAUUGUCUUCCUU; Dharmacon) were optimized for gene-silencing efficiency and used between 5 and 20 nmsingly or in a 5 nm(total concentration) pool.

### Time-lapse microscopy

Time-lapse recordings of HeLa YFP–tubulin cells were made using a DeltaVision Spectris microscope (Applied Biosystems) fitted with 37°C chamber. YFP –tubulin images were collected with a ×60 1.4 numerical aperture objective, as a series of 12 × 1 μmZ-stacks at 5 or 10 min intervals, together with a single differential interference contrast microscopy (DIC) reference image. Fluorescence was viewed as projections of maximum pixel intensities of each stack onto a single plane and exported in 16-bit TIFF format using ImageJ(National Institutes of Health). DIC images were used to determine the time of nuclear envelope break down and of the onset of anaphase.

### EGFR degradation assay

Mock -transfected HeLaM cells or HeLaM transfected with spastin siRNA were serum starved overnight and then treated with 100 ng/mL of EGF (Calbiochem) in the presence of cycloheximide (10 μg/mL). At selected time-points, cells were washed with cold PBS and then harvested into 1× Laemmli sample buffer. Samples were run on SDS–PAGE and immunoblotted to detect EGFR. EGFR band density was quantified using a Geldoc GS-710 densitometer and Quantity Onesoftware (Bio-Rad). Density values were normalized versus those of the corresponding unstimulated sample, and the results were analysed in GraphPad Prism5.01 for Windows (GraphPad Software).

### ts 045-VSVG-GFP assays

HelaM cells were cotransfected with 60 or 68 kD wild-type or ATPase-defective K388R myc-spastin constructs and ts045 VSVG-GFP. Twenty-four hours post transfection cells were incubated at 40°C for 16 h. For 0 min time-point, cells were fixed at 40°C, and for all subsequent time-points, cells were incubated in media preconditioned to 32°C and fixed at the same temperature. Following the temperature shift, cells were processed for immunofluorescence as previously described or used for endo H digestion assays. Coverslips were manually scanned for cotransfected cells, and individual cells were counted. Histograms and statistical analyses were made using GraphPad Prism.

## Supporting Information

**Figure S1: Verification of spastin antibodies and spastin expression pattern.** Two spastin antibodies were used in this study, spastin 3G11/1 and spastin86-340. Both were raised against epitopes present in the 60 and 68 kD spastin isoforms (Figure 1). A) Immunoblot of mock-transfected Hela cells or HeLa cells transfected with pooled spastin siRNA oligonucleotides 1–4, blotted using spastin86–340 antibody. Sixty-eight, 64, 60 and 55 kD bands are visible in the mock-transfected sample, while the 60 and 55 kD bands are lost following siRNA-mediated spastin depletion. Similar effects were found when the siRNA oligonucleotides were used individually. Actin blotting is shown to verify equal loading. B) Immunoblot of mock-transfected Hela cells or HeLa cells transfected with spastin siRNA, blotted using spastin 3G11/1 antibody. For immunoblotting, 3G11/1 was less sensitive than the spastin86-340 antibody, but it was able to detect the strong 60 kD band. Actin blotting verifies equal loading. C) Immunoblots using spastin86–340 in the cell lines indicated. D and E) In HeLa cells transfected with pooled spastin siRNA oligonucleotides 1–4 (E) and labeled with anti-spastin 3G11/1, spastin labeling is lost compared with mock-transfected HeLa cells (D). Images taken with identical confocal microscope settings. Asterisks in (D) indicate cells showing nuclear spastin labeling. Spastin86–340 did not specifically detect endogenous spastin under immunofluorescence. F) Hela cells labeled with anti-spastin 3G11/1 occasionally showed filamentous structures, labeled by arrowheads in the inset box. Scale bars in these and all subsequent micrographs = 10 μm. In these and all subsequent supplementary figures, images were obtained from cells fixed with formaldehyde.

**Figure S2: Endogenous spastin labeled with selected markers.** A–C) Hela cells labeled with antibodies to spastin (A) and mannose 6 phosphate receptor (M6PR), a marker of late endosomes (B). There is minimal colocalization (C). D–F) Endogenous spastin (D) was recruited to VPS4E235Q-positive endosomes (E) in transiently transfected NSC34 cells.

**Figure S3: Localization of spastin isoforms at midbody.** A–C) Hela cells transfected with 60 kD myc-spastin (A) show colocalization with alpha-tubulin (B) in the midbody. D–F) Sixty-eight kiloDalton myc-spastin (D) shows partial colocalization with alpha-tubulin (E) in the midbody of HeLa cells.

**Figure S4: Sixty kiloDalton spastin is not localized to the early secretory pathway.** Hela cells were transfected with 60 kD myc-spastin **(**A–C) or 60 kD myc-spastinK388R (D–F). There was no colocalization between 60 kD myc-spastin (A) or 60 kD myc-spastinK388R (D) and calreticulin (B and E). Note also that although 60 kD myc-spastinK388R does bundle MTs, it does not cause redistribution of the ER, unlike 68 kD myc-spastinK388R.

**Figure S5: ATPase-defective 68 kD spastin but not 60 kD spastin delays ER–Golgi traffic.** HeLa cells were cotransfected with VSVG-GFP and 68 kD myc-spastin (A–H), 68 kD myc-spastinK388R (I–P), 60 kD myc-spastin (Q–T) or 60 kD myc-spastinK388R (U–X). VSVG-GFP was then released in a pulse from the ER, and cells were examined at the time-points indicated. A–D) In cells expressing 68 KD myc-spastin (A), just after VSVG-GFP release, some VSVG-GFP puncta (B) are beginning to emerge from the ER (C) and these colocalize with 68 kD myc-spastin (arrowheads). E–H) At 120 min after release, VSVG-GFP (F) is strongly associated with the plasma membrane (note membrane ruffles in F). Sixty-eight kiloDalton myc-spastin expression (E) remained unchanged from earlier time-points, appearing as cytosolic puncta. I–L) In Hela cells transfected with 68 kD myc-spastinK388R (K), there was a complete redistribution of the ER (I) onto tubular structures that also contain bundled MTs (data not shown). Just after shifting to the permissive temperature, these tubules label strongly with VSVG-GFP (J). M–P) At 120 min following the temperature shift, the colocalization between 68 kD myc-spastinK388R (M) and VSVG-GFP (N) seen at earlier time-points is lost and there is partial colocalization between VSVG-GFP and GM130 (O) (inset). Plasma membrane fluorescence is minimal. Q–T) In cells expressing 60 kD myc-spastin (Q), 10 min after release from the ER, VSVG-GFP (R) strongly colocalized with the Golgi marker GM130 (S; arrowheads). U–X) Expression of 60 kD myc-spastinK388R resulted in a cellular phenotype of thickened and bundled MTs in higher expressing cells (U), with diffuse and punctate cytoplasmic staining in lower expressing cells (data not shown). At 10 min after release from the ER, VSVG-GFP (V) was strongly colocalized with the Golgi marker GM130 (W; inset).

**Figure S6: Sixty-eight kiloDalton myc-spastinΔMTB-K388R colocalizes with the ER.** A–C) Transiently expressed 68 kD myc-spastinΔMTB-K388R (A) colocalized with the ER marker calreticulin (B) in HeLa cells.

**Figure S7: Depletion of spastin has no effect on EGFR degradation.** A) A representative EGFR degradation assay. Mock-transfected HeLa cells or HeLa cells transfected with spastin siRNA were serum starved overnight and then stimulated with 100 ng/mL of EGF in the presence of cycloheximide. Cells were harvested at the indicated time-points and immunoblotted versus spastin, using spastin86-340 antibody, or EGFR. Actin blotting is shown to verify equal loading. B) EGFR immunoblots were quantified by densitometry in six independent experiments carried out as in (G). Error bars represent 95% confidence intervals.

**Figure S8: Spastin depletion by four individual siRNA oligonucleotides delays completion of cytokinesis.** A) Representative experiments showing cumulative timings of the cytokinesis-associated MT disruption event in mock-transfected YFP–tubulin HeLa cells or YFP–tubulin cells transfected with individual spastin siRNA oligonucleotides 1, 2, 3 or 4. Timings were obtained as described in Figure 8. Spastin depletion with each oligonucleotide caused a delay in the midbody MT disruption event after anaphase. A representative immunoblot showing the effect of the individual siRNAs on endogenous spastin is shown. The 60 kD spastin isoform is effectively depleted by all four oligonucleotides. However, oligonucleotide 2 (whose target sequence is in spastin exon 4) does not deplete the 55 kD spastin isoform (asterisks), and interestingly, this oligonucleotide had the smallest effect in delaying the MT disruption event. p ≤ 0.0001 for timings after treatment with each oligo compared with timings from mock-transfected cells (Student’s *t*-test). Number of cells analysed: mock = 43, siRNA1 = 12, siRNA2 = 45, siRNA3 = 44 and siRNA4 = 11. B) Representative experiments showing cumulative timing of anaphase onset in mock-transfected YFP–tubulin HeLa cells or YFP–tubulin cells transfected with individual spastin siRNA oligonucleotides 1, 2, 3 or 4. Time from nuclear envelope breakdown (NEBD) to separation of duplicated chromosomes at anaphase was measured. Number of cells analysed: mock = 39, siRNA1 = 6, siRNA2 = 36, siRNA3 = 52 and siRNA4 = 10. C) Time-lapse series of mock-transfected HeLa YFP–tubulin cells to illustrate how timings of NEBD and anaphase were determined. Both DIC imaging and the distribution of YFP–tubulin fluorescence reveal NEBD between the 5 and the 10 min time-points in this series. Anaphase is measured as the point of separation of duplicated chromosomes seen by DIC, or the elongation of the mitotic spindle seen by YFP–tubulin fluorescence, seen here at the 30 min time-point.

**Video S1:** Movie of HeLa cells stably transfected with YFP–tubulin undergoing cytokinesis. The cell on the left is a control cell, whereas the cell on the right is from a population depleted of spastin by siRNA. These cells correspond to time-lapse images shown in Figure 8.
