# Supplementary figures and images for: Spastin Couples Microtubule Severing to Membrane Traffic in Completion of Cytokinesis and Secretion
Source: Traffic. 2008 Oct 29;10(1):42–56. doi: 10.1111/j.1600-0854.2008.00847.x (PMC2709849; doi:10.1111/j.1600-0854.2008.00847.x)

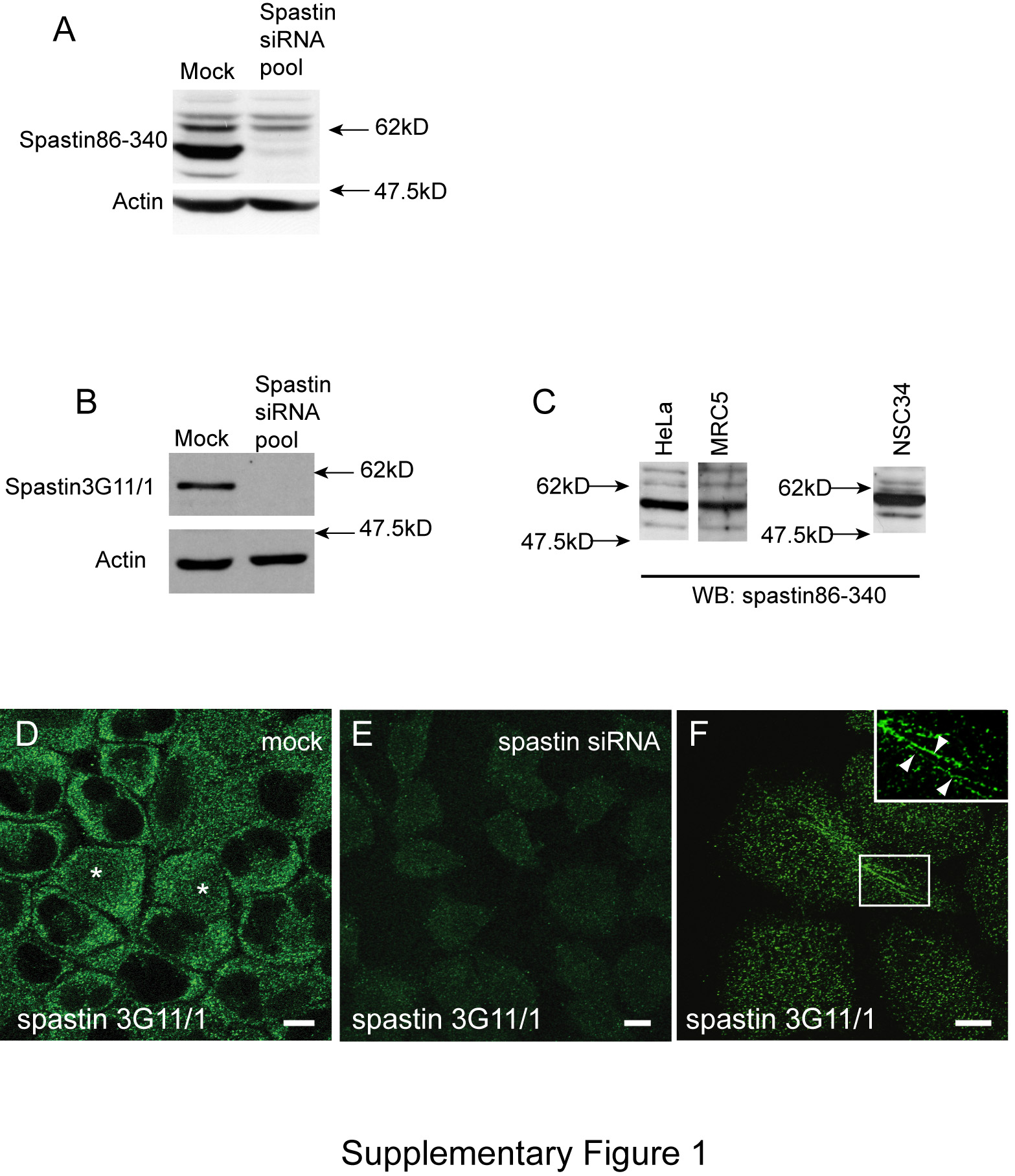

Supplement: Supplementary file 1 [file tra0010-0042-SD1.jpg]

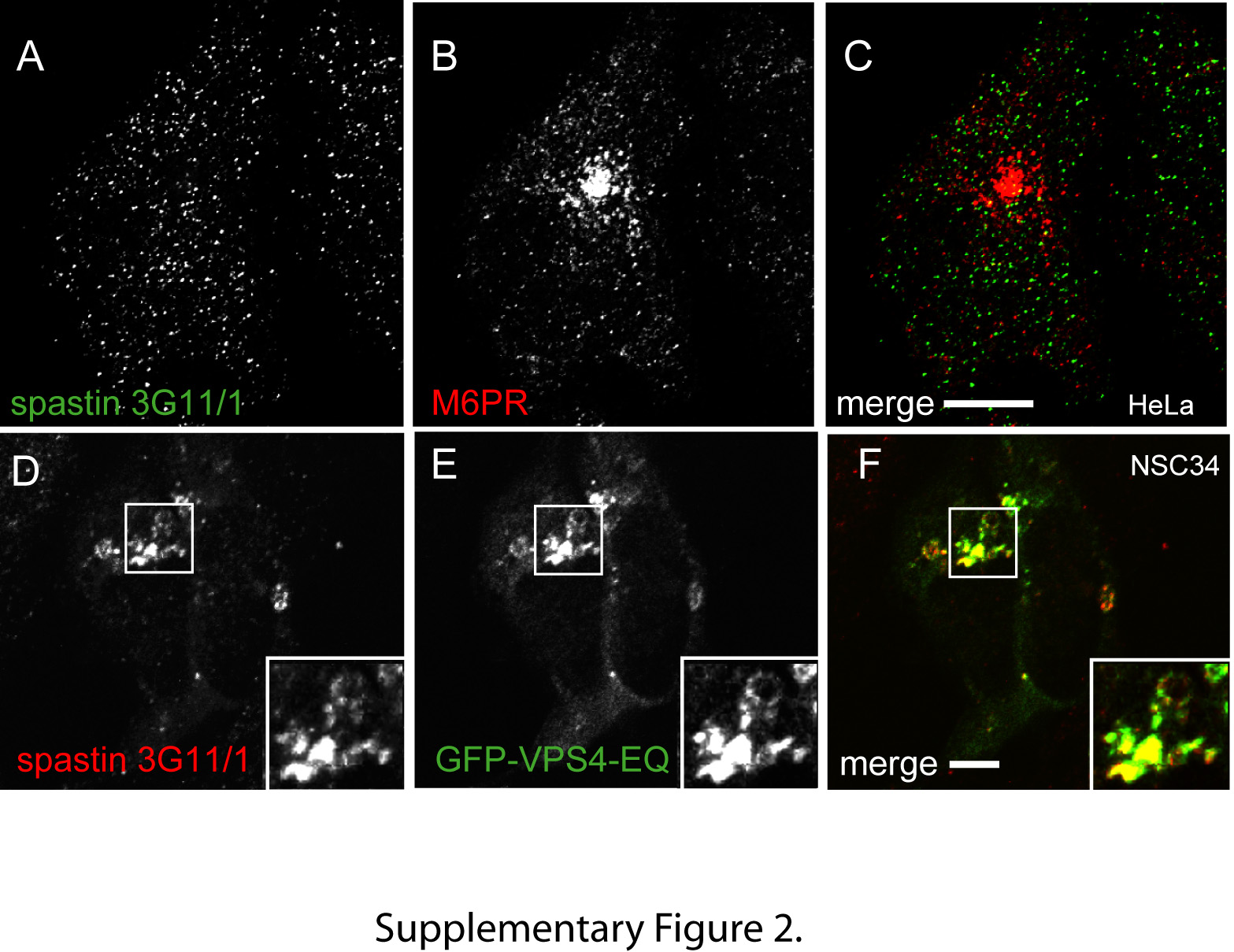

Supplement: Supplementary file 2 [file tra0010-0042-SD2.jpg]

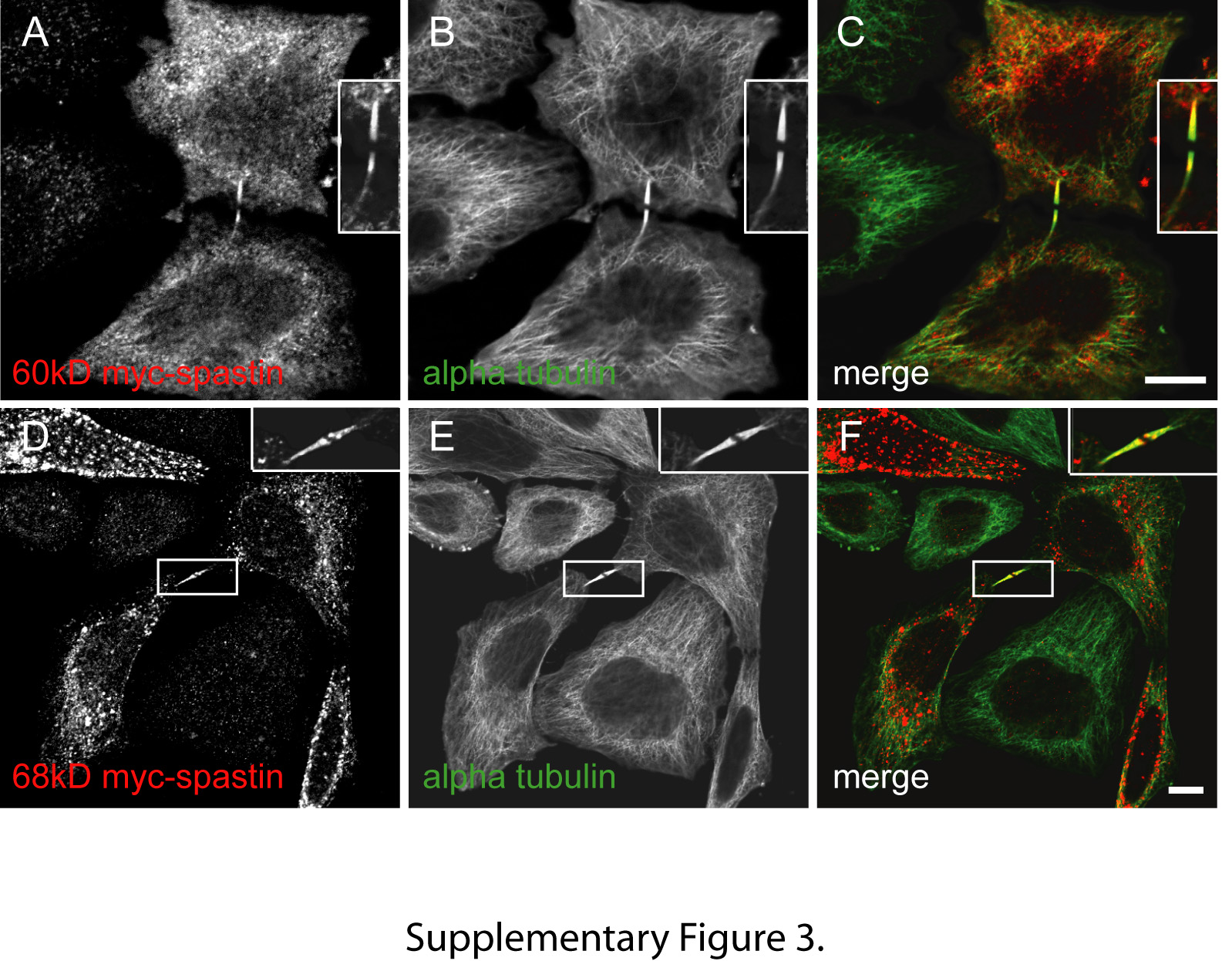

Supplement: Supplementary file 3 [file tra0010-0042-SD3.jpg]

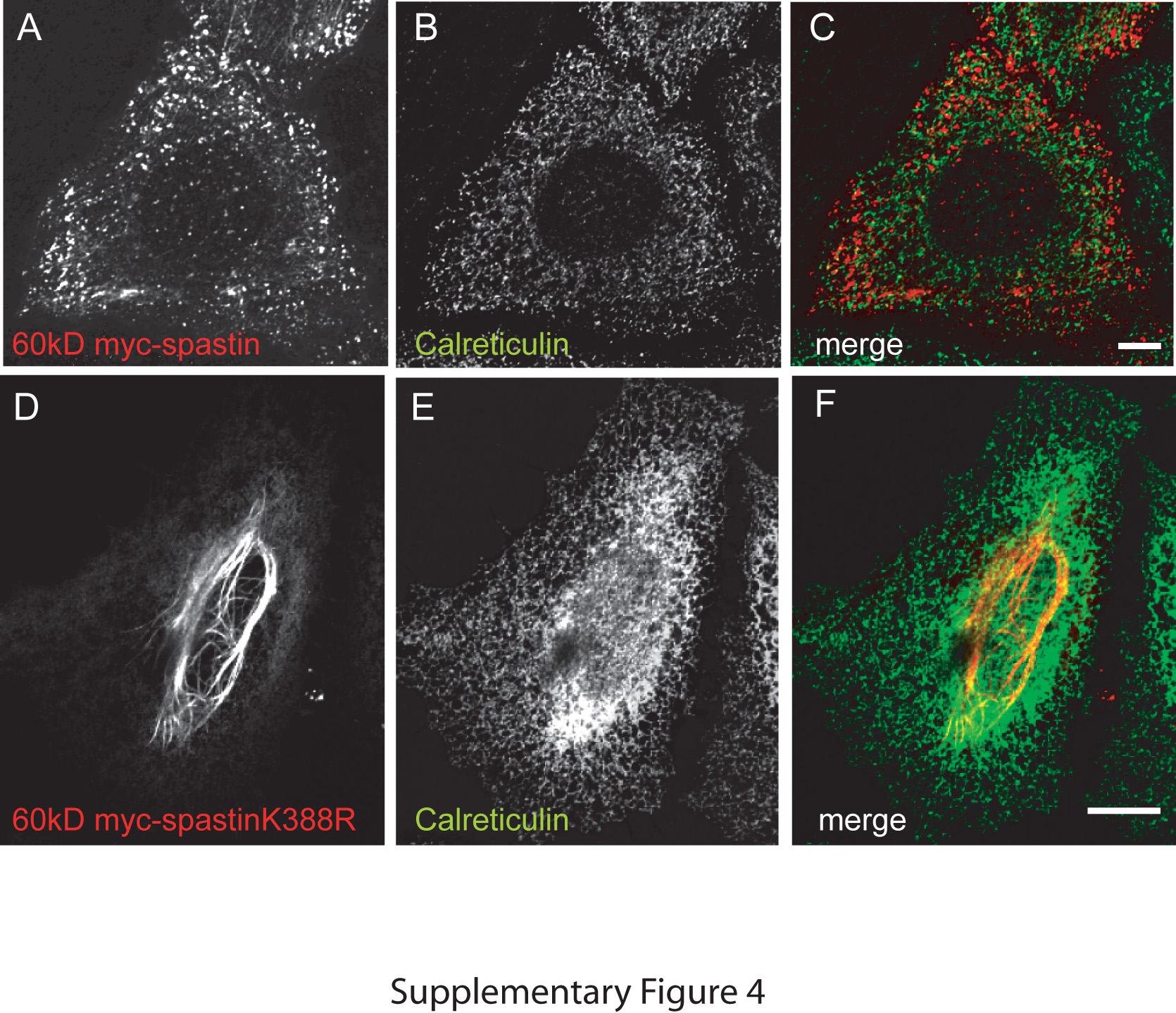

Supplement: Supplementary file 4 [file tra0010-0042-SD4.jpg]

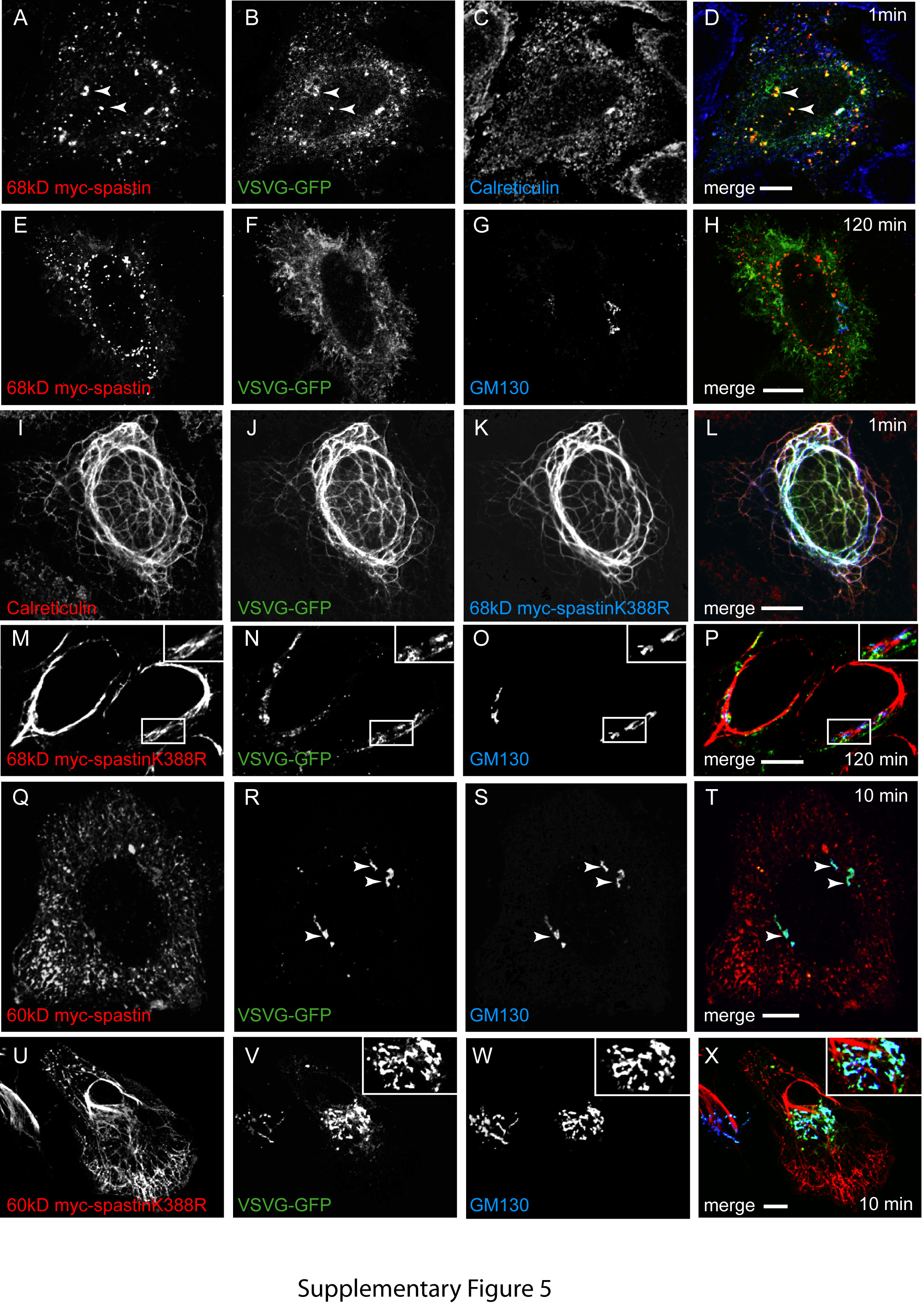

Supplement: Supplementary file 5 [file tra0010-0042-SD5.jpg]

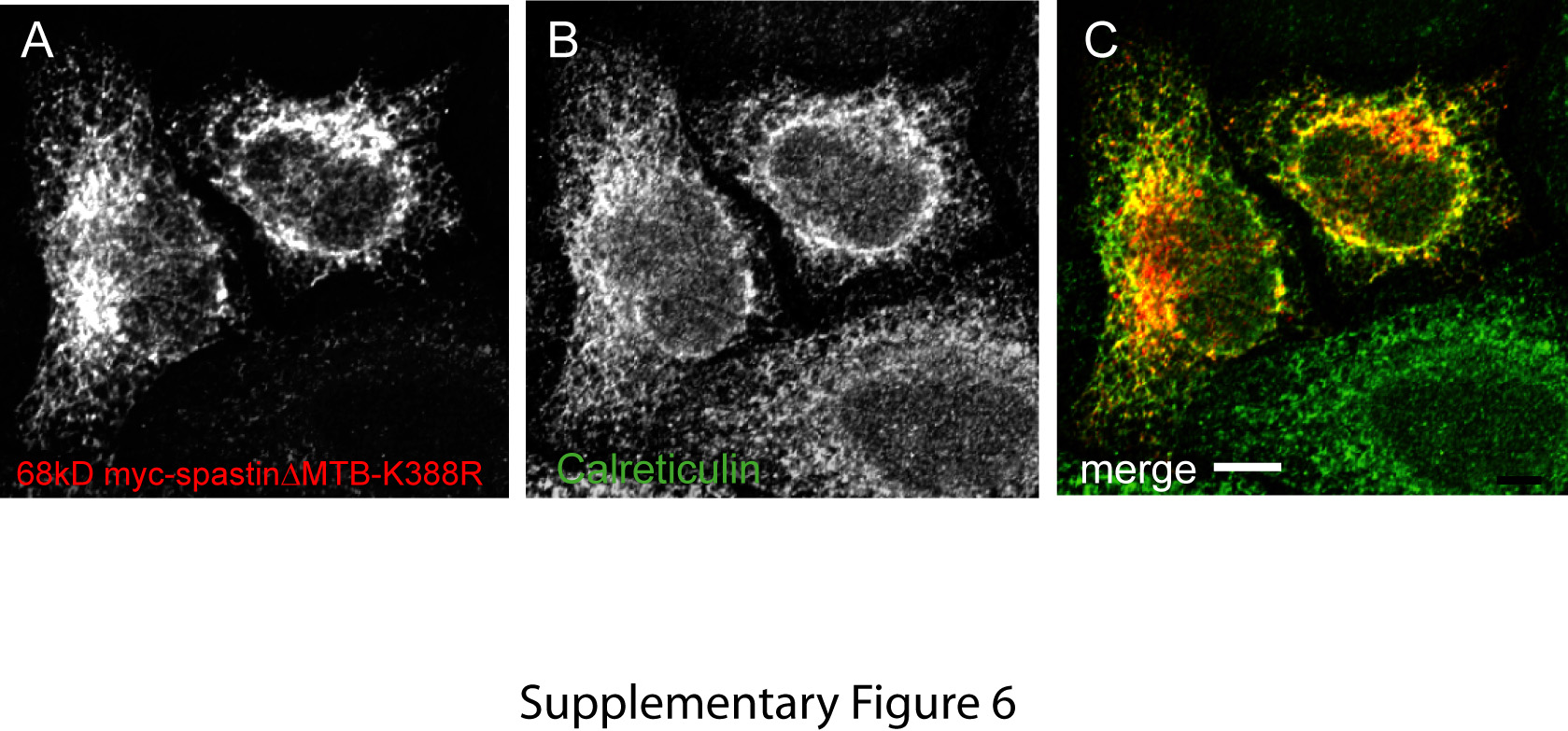

Supplement: Supplementary file 6 [file tra0010-0042-SD6.jpg]

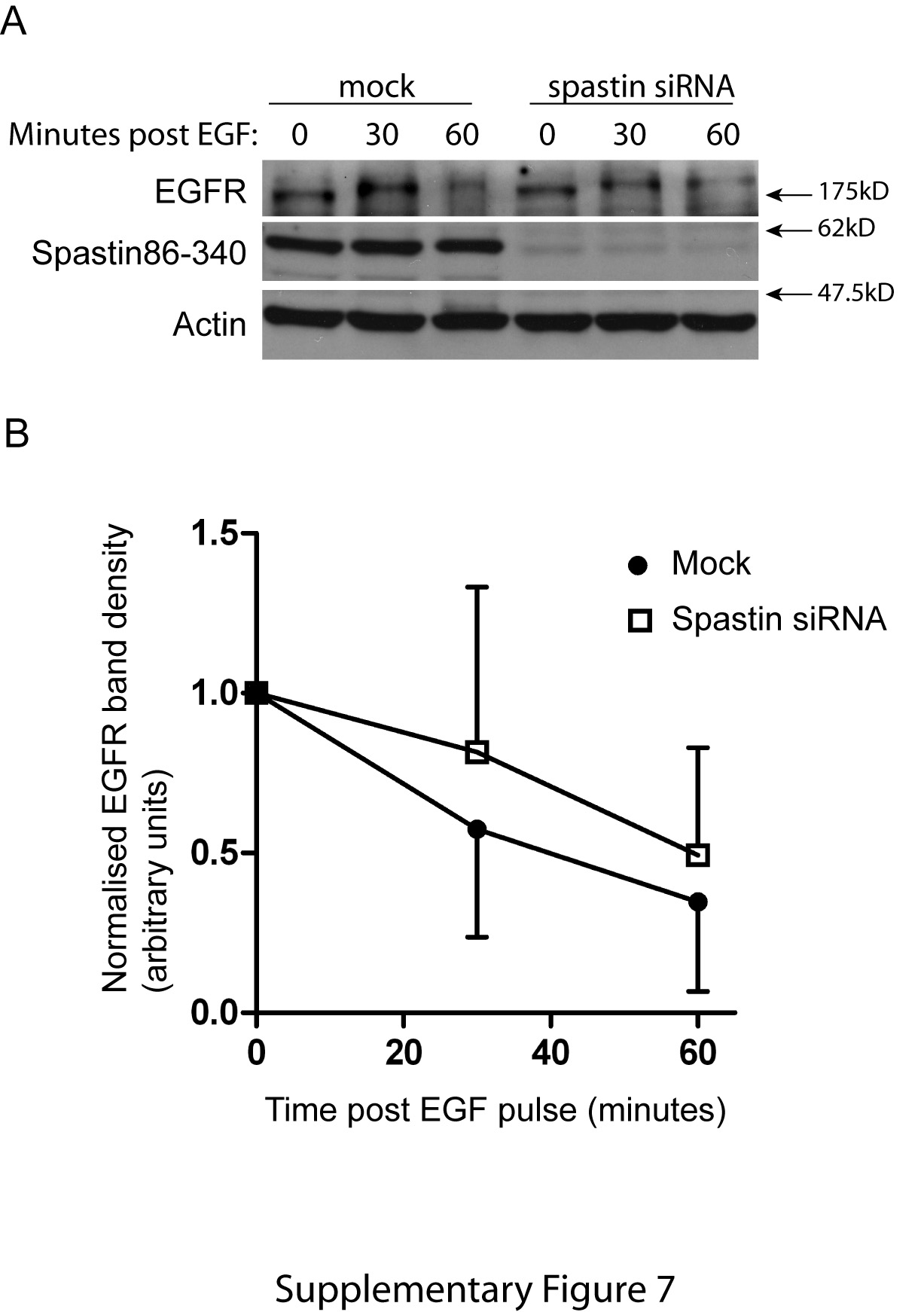

Supplement: Supplementary file 7 [file tra0010-0042-SD7.jpg]

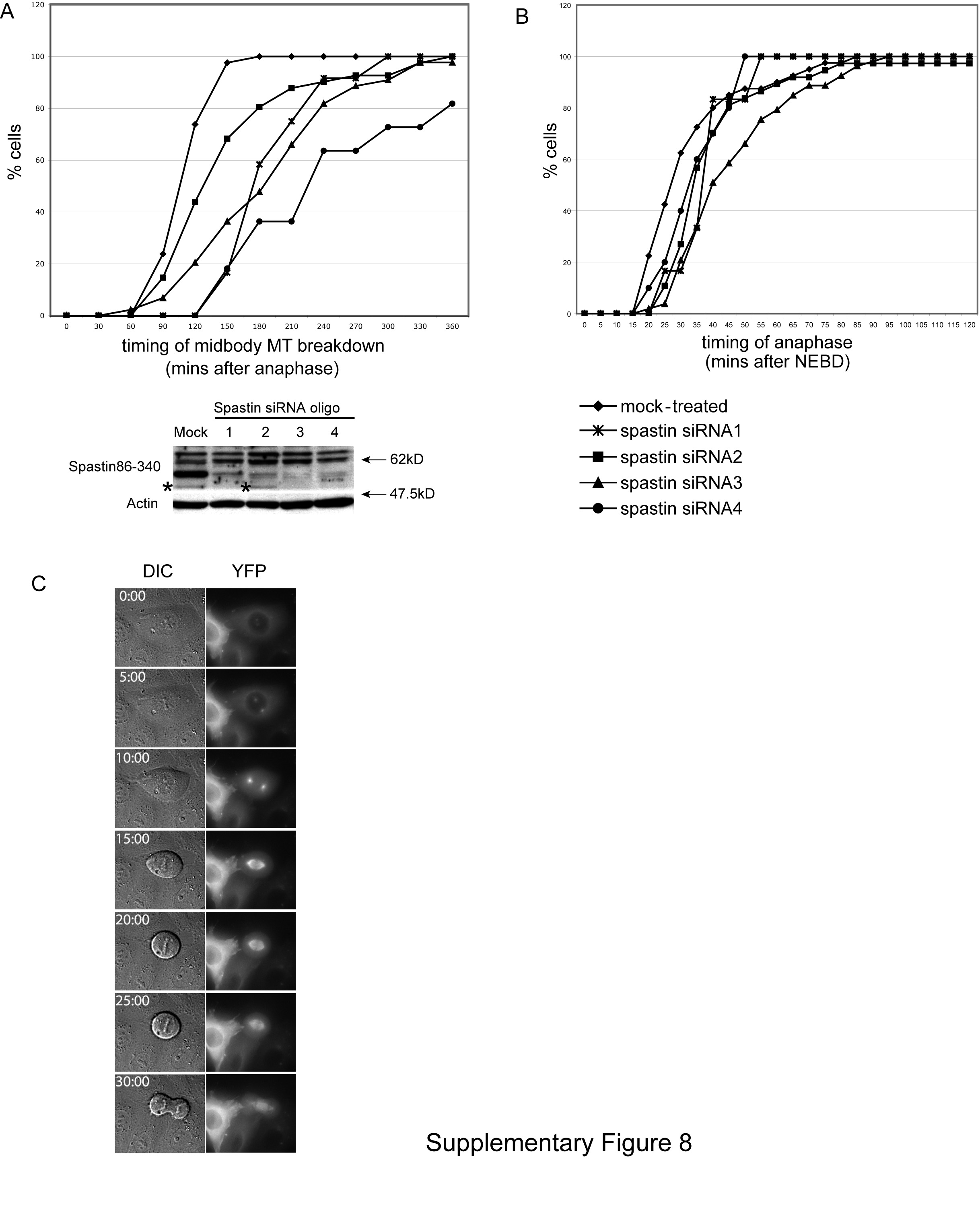

Supplement: Supplementary file 8 [file tra0010-0042-SD8.jpg]
